# The Noradrenergic Brain in Parkinson’s Disease

**DOI:** 10.1007/s11910-026-01499-x

**Published:** 2026-06-16

**Authors:** Jacopo Pasquini, Giovanni Palermo, Nicola Pavese, Roberto Ceravolo

**Affiliations:** 1 Department of Clinical and Experimental Medicine, University of Pisa, Pisa, Italy; 2 Translational and Clinical Research Institute, Faculty of Medical Sciences, Newcastle University, Newcastle Upon Tyne, UK; 3 Department of Nuclear Medicine, Institute of Clinical Medicine Aarhus University, Aarhus, Denmark; 4 Neurodegenerative Diseases Center, Azienda Ospedaliero Universitaria Pisana, Pisa, Italy

**Keywords:** Locus coeruleus, Parkinson’s disease, Noradrenergic, Neuromelanin, Cognitive impairment, Tremor

## Abstract

**Purpose of Review:**

Cerebral noradrenergic activity modulates physiological functions of behaviour, cognition, movement, arousal and sleep. This review aims to provide an accurate summary of the current knowledge on the involvement of the noradrenergic system in Parkinson’s Disease (PD) and its clinical correlations based on neuroimaging studies.

**Recent Findings:**

Studies in PD highlight neuromelanin MRI signal loss in the locus coeruleus (LC), and positron emission tomography shows noradrenergic denervation across subcortical and cortical areas. More severe phenotypes of PD, manifesting with cognitive decline, apathy, REM sleep behaviour disorder and autonomic dysfunction, are associated with more severe noradrenergic dysfunction. Conversely more preserved noradrenergic transmission is common in tremulous PD. Furthermore, noradrenergic dysfunction, is also involved in transient motor manifestations such as tremor and freezing of gait.

**Summary:**

Recent neuroimaging advances greatly expanded the knowledge about noradrenergic dysfunction pathophysiology in PD. However, pharmacological treatment of its several associated manifestations is still lacking and needs further investigation.

## Introduction

The noradrenergic system modulates several homeostatic processes such as behavioural and cognitive processes, arousal and sleep. Prefrontal-dependent attention and working memory, long-term memory and synaptic plasticity, emotional-dependent memory, behavioural responses to stress, goal-directed behaviour and sensory processes are complex functions involving cortical and subcortical structures that are critically modulated by the noradrenergic system [1–7]. These processes are influenced not only by noradrenergic neuronal responses, but also by direct effects of noradrenaline on glial and blood vessels cells [8, 9]. Additionally, noradrenergic input to the motor cortex facilitates movement initiation and execution via adrenergic receptor actions on pyramidal neurons [10] and descending noradrenergic pathways to the brainstem and spinal cord increase motoneuron excitability and influence posture and locomotion [11].

The identification of noradrenergic degeneration in Parkinson’s Disease (PD), with neuropathological changes in the locus coeruleus (LC) similar to those found in the substantia nigra (SN), dates back to almost 100 years ago [12–14]. Subsequent studies provided thorough neuropathological details highlighting the presence of Lewy bodies in this region [15–19].

In more recent years, the integrity and function of the brain noradrenergic system in PD have been investigated through molecular and structural neuroimaging. Overall, neuropathological and MRI studies have provided evidence of a heterogeneous involvement of the LC in PD, with greater neuronal loss affecting more severe phenotypes. Indeed, greater involvement of the noradrenergic system is associated with the presence of more severe cognitive and behavioural manifestations, REM sleep behaviour disorder (RBD), and orthostatic hypotension. Conversely, a relatively more preserved noradrenergic function may be associated with manifestation of parkinsonian tremor.

Although a significant amount of knowledge has been collected regarding noradrenergic function in vivo in PD, knowledge gaps remain regarding clinical correlations and more importantly, treatment of noradrenergic dysfunction.

In this review, the state of the noradrenergic system and its clinical correlations in PD will be described.

## Search Strategy

Pubmed/MEDLINE database was interrogated, including results up to July 2025. Search words (across all fields) were “noradrenaline”, “locus coeruleus” and “parkinson” and either “MRI” or “PET”. Articles written in English investigating noradrenergic function in humans were selected; additional articles with these characteristics were extracted from the references.

## Neuroanatomical Organization of the Central Noradrenergic System

The main noradrenergic neurons of the brain are located in the LC (A6 cell group) in the dorsal pons, which provides the majority of noradrenaline to the forebrain[20]. Additional noradrenergic cell groups, termed A1–A7, are distributed in the medulla and pons and contribute to autonomic and motor regulation.[21] The A1 and A2 nuclei in the ventrolateral medulla and nucleus tractus solitarius regulate cardiovascular and visceral functions.[22] The A5–A7 pontine groups project to the spinal cord and brainstem, influencing pain modulation and sympathetic output.[23] Together, these nuclei form the central noradrenergic system with widespread projections throughout the brain and spinal cord [11]

**Fig. 1:**
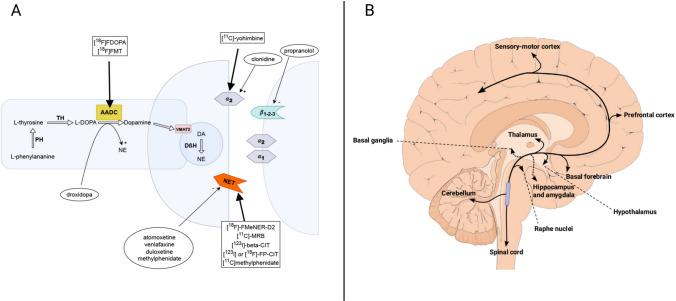
**A**) Schematic representation of a central noradrenergic synapse. Compounds displayed in squares are examples of radioligands; compound displayed in circles are examples of drugs (the plus “ + ” symbol indicate and activatory effect on the target, the minus “-” symbol indicates an inhibitory effect); **B**) Schematic representation of locus coeruleus efferent pathways. The blue rectangle depicts the locus coeruleus approximate location. Abbreviations. α_x_, β_x_: norepinephrine receptors subtypes; AADC: L-aromatic aminoacid decarboxylase; DβH: Dopamine beta hydroxylase; DA: dopamine; NE: norepinephrine; NET: norepinephrine transporter; PH: phenylalanine hydroxylase; TH: tyrosine hydroxylase; VMAT2: Vesicular monoamine transporter 2. *Radioligands displayed in the figure*: β-CIT: 2β-carbomethoxy-3b-(4-iodophenyl)tropane. FDOPA: 6-Fluoro-L-dopa; FMeNER-D2: α-(2Fluoro[2H2]methoxyphenoxy)phenoxy)benzyl)morpholine; FMT: 6-Fluoro-L-m-tyrosine; FP-CIT: Fluoropropyl-carbomethoxy-3b-(4-iodophenyl)tropane; MRB: Methylreboxetine. Fig. 1B was adapted from Servier Medical Art (https://smart.servier.com), licensed under CC BY 4.0 (https://creativecommons.org/licenses/by/4.0/)

Around 15–30,000 thousand neurons form the thin, elongated (length 14.5 mm, width 2.5 mm) LC groups on either side of the fourth ventricles. In fresh neuropathological specimens, these two small groups appear blue owing to the presence of (neuro)melanin produced and accumulated in the catecholamine synthesis pathway (Fig. 1A). Current literature suggests the presence of well-segregated intra-LC neuronal groups that give rise to organized axonal outputs and targets, and other neuronal groups with divergent widespread innervation targets. Axon terminals emerging from functionally-related LC neuronal groups have widespread collaterals distributed in a coordinated manner onto functionally-related circuits[1, 24]. This efferent noradrenergic system is spread throughout the central nervous system, from the neocortex to the spinal cord (Fig. 1B). This organization mediates LC physiological functions: noradrenergic input to thalamus and cortex influences arousal and behaviour, input to prefrontal cortex modulates working memory, attention and set-shifting, inputs to sensory thalamus and cortices influences sensory processing across all modalities, projections to the hippocampus and amygdala modulate synaptic plasticity and memory consolidation [25]. Interestingly, it is generally thought that the basal ganglia are devoid of LC noradrenergic terminals[20]; however, a small body of evidence showed that thin, sparse axonal projections are likely present in the caudate and putamen and may be involved in the regulation of the striato-motor network [26, 27].

Afferent modulation to the LC is thought to come from forebrain regions (prefrontal areas, amygdala, hypothalamus), brainstem nuclei and cerebellar Purkinje cells [1, 25, 28–30]. The nucleus is organized with a cell-body containing core and a shell where dendrites ramify. LC activity is modulated differentially by afferents through synapses with either the core or the peripheral areas; however, the precise mechanisms remain to be elucidated [1, 20].

The effect of noradrenaline on target neuronal and non-neuronal populations is exerted through two different sets of receptors, α- and β-receptors. While α_1_- and β-receptors are commonly post-synaptic and have excitatory effects on the target cell, α_2_ are pre-synaptic autoreceptors that inhibit the further release of noradrenaline from the axon terminal. A specific noradrenaline transporter (NET) reuptakes noradrenaline into the releasing neuron. The main noradrenergic markers (and their molecular neuroimaging ligands) are summarized in Fig. 1A.

## Clinical Investigation of the Central Noradrenergic System

In vivo investigation of the noradrenergic system can be performed mainly through neuroimaging, i.e. MRI and molecular imaging modalities (positron emission tomography, PET, and single photon emission tomography, SPECT).

MRI can provide useful information about the structural integrity of the LC. Indeed, different sequences (magnetisation prepared, either T1 turbo spin echo or gradient echo), can exploit the paramagnetic properties of neuromelanin to produce a hyperintense signal [31–33]. This signal is commonly attributed to the LC, given its well-known neuropathological content of neuromelanin, a neuronal-specific melanin pigment that binds to metal ions. Furthermore, a close spatial and quantitative correlation between LC-associated MRI contrast and neuromelanin content in neuropathological examinations has also been shown [34, 35]. Neuromelanin is a complex macromolecular structure composed of melanin bound to proteins, lipids, iron and copper [36]. The exact molecular mechanism responsible for the hyperintense signal in neuromelanin-rich structures has been investigated thoroughly due to its complex mechanisms. It is hypothesized to result from both neuromelanin and other environmental factors such as the presence of elevated free water content [32]. A summary of current understanding of neuromelanin biological and imaging properties is beyond the scope of this review and can be found in *Betts *et al.[37]

It is noteworthy that while neuromelanin-sensitive MRI can detect LC neuromelanin loss, this is not necessarily reflective of the overall axon terminals integrity. In neurodegenerative disorders, a dying-back mechanism has been shown in several nuclei, with a disproportionate loss of axons compared to cell bodies (e.g. substantia nigra dopaminergic terminals, nucleus basalis of Meynert cholinergic terminals). Evidence of a similar phenomenon in the noradrenergic system in PD comes from one *in-vivo* neuromelanin MRI and ^11^C-MeNER PET that found that loss of NET in subcortical areas exceeded on average 10–20% the loss of LC neuromelanin in PD compared to controls. Furthermore, no correlation was present between LC neuromelanin content and NET availability in the same areas [38, 39].

Some recent studies also highlighted the possibility of investigating LC activity through functional MRI (fMRI), with or without the help of neuromelanin localization of the LC [40, 41]. Further studies are needed to elucidate whether this type of investigation can provide additional information about LC functions in vivo.

Molecular imaging of the noradrenergic system is possible mainly through PET. However, the limited number of selective, brain-penetrant ligands to noradrenergic-specific targets currently limits these investigations. Figure 1A displays the main noradrenergic PET ligands available to investigate the central noradrenergic system. PET also allows the investigation of the specific catecholaminergic activity in noradrenergic neurons contained within the nucleus through ^18^F-DOPA, which is metabolized to ^18^F-dopamine by aromatic aminoacid decarboxylase (AADC) and enters the noradrenaline biosynthetic pathway [42, 43]. PET and SPECT presynaptic ligands that bind to the dopamine transporter (DAT) such as FP-CIT have some affinity also for the serotonin transporter and to a lesser extent for the NET (with a ratio of about 1:70 compared to the DAT[44, 45]). Due to the diffuse presence of dopaminergic and serotonergic terminals, its use as a NET transporter ligand is limited, but it has been used for the semiquantitative estimation of NET availability in the locus coeruleus [46–48].

Indirect measures of the noradrenergic system activity may also be collected. Historically, two of the most common measures employed are pupil diameter and heart rate recordings, with the first being strongly correlated with LC activity in primate animal models[10, 41]. Such proxy measures can be recorded in translational and clinical research experiments to acquire a continuous estimation of the LC noradrenergic activity.

## The Central Noradrenergic System Integrity in Healthy Aging and PD

Since the advent of neuromelanin-sensitive MRI, several studies investigated LC neuromelanin signal in healthy subjects across lifespan. LC neuromelanin increases from early life into adulthood, a phenomenon that was described in neuropathological and in vivo MRI studies. Instead, conflicting evidence exists about the trajectory in older adults, where both stable and decreasing trajectories were shown[36, 49–51]. MRI studies reported an inflection point around the age of 60 with a subsequent decrease in neuromelanin-related signal [52–55]. As suggested by the great amount of preclinical evidence regarding LC involvement in cognitive processes, studies in vivo in humans recently revealed several aspects of this associations. In particular, several studies highlighted that the reduction in LC neuromelanin signal in older adults is associated with declining overall cognitive and memory performances, [56, 57] with stronger correlations with the rostral LC projecting to forebrain structures. Conversely, in cognitively unimpaired subjects (tested 1 year apart) between 60 and 80 years old, LC neuromelanin was not associated with age [51]. A more recent study integrated a wide cognitive assessment with both LC and SN neuromelanin signal investigation in young and older adults (the latter with age range 60–80 years of age). A stronger link between LC integrity and episodic memory was found, as opposed to the association between SN and working memory [58]. Interestingly, after a 2-year follow up, LC neuromelanin signal decline showed a significant association, stronger than those of age and education, with declining episodic memory.

Evidence from molecular imaging studies in healthy ageing is limited. The NET tracer may be targeted by reboxetine derivatives, e.g. carbon or fluorine methylreboxetine or MeNER-D2. These tracers show high NET concentration in areas known to be rich in noradrenergic terminals from animal and pathological studies, i.e. LC, midbrain and pontine raphe, red nucleus, thalamus and hypothalamus, with lower concentrations in basal ganglia and occipital cortex. In controls, this tracer showed inverse correlations with age in several brain areas (including LC and hypothalamus), with higher binding potential in younger subjects [59].

Notably, the fluorinated MeNER compound has shown potential also for the investigation of cortical binding, despite the ^18^F spillover to the skull which represents a limitation for cortical NET quantification [60]. ^11^C-yohimbine may be used to investigate α_2_ autoreceptors on noradrenergic terminals. In healthy subjects, highest areas of binding are cortical areas and hippocampus, with moderate binding in subcortical structures (thalamus, caudate nucleus, putamen, and amygdala) [61].

### The Integrity of the Noradrenergic System in PD

In PD, post-mortem LC cell count reductions were shown in pathological studies [12, 14], with heterogeneity in cell loss, ranging from mild to severe (30 to 90%) [17, 62]. Cell loss was shown to be present throughout the rostrocaudal extent of the LC in PD brains (without significant differences), as opposed to the pattern seen in Alzheimer’s Disease (AD), where moderate-severe rostral cell loss is usually present in the absence of significant cell loss in the caudal LC [17]. However, in PD dementia, more severe rostral cell loss was reported [62]. Accordingly, reduction of noradrenaline or related biosynthetic enzymes (e.g. dopamine-β-hydroxylase, the converting enzyme that transforms dopamine into noradrenaline) were shown in several brain areas, including the ventral tegmental area and nucleus accumbens, the hypothalamus, the thalamus, cerebellum and motor cortex [63–68].

*In-vivo* LC neuromelanin MRI signal reduction was shown in several studies in PD compared to controls.[33, 38, 69–75], as reported in a systematic review [76]. In some studies, greater reductions were shown in PD patients with REM sleep behaviour disorder compared to those without [39, 69, 77]. Two studies conducted with 7 T MRI reported greater neuromelanin signal loss in the middle and caudal parts of the LC in PD compared to controls [78, 79], a finding that was also shown with 3 T MRI [38, 80].

Several studies have reported the lack of association between LC signal loss and PD duration. Left–right asymmetries in LC neuromelanin signal loss were also reported. However, it is questionable whether these differences reflect true changes or are caused by MRI field inhomogeneities [54, 69, 78], since findings in pathological studies show a symmetrical distribution of neuromelanin containing cells[49, 81, 82]

A few molecular imaging studies have investigated the state of noradrenergic terminals in PD. Reduction of NET availability in PD (regardless of their RBD status) compared to controls was shown in thalamus, hypothalamus, red nucleus and raphe nuclei. [38, 39, 83, 84] In patients with PD and RBD, the reductions were more pronounced and also involved the LC. Interestingly, in PD without RBD, no significant reductions were found compared to controls in LC and raphe nuclei [39]. Compared to controls, PD patients also showed reduced NET availability in the primary motor and sensory cortices, with more severe reductions in patients with greater motor severity [85]. Another study investigated NET availability in PD patients with and without tremor: ^11^C-MeNER was reduced in thalamus, red nucleus, LC and raphe nuclei in patients without tremor compared to controls, but only in the dorsal raphe in patients with tremor. Furthermore, binding in the LC in the tremulous group was similar to that of controls [86]. These findings suggest a less pronounced LC degeneration in tremulous PD, with a possible initial dying-back mechanism of noradrenergic terminals in the serotonergic dorsal raphe nuclei.

Studies investigating the α_2_ noradrenergic receptor are even scarcer. One study in 30 PD patients investigated ^11^C-yohimbine binding in the cortex, basal ganglia and thalamus and found reduced binding in PD compared to controls in motor cortex, parieto-occipital areas, insula and putamen. Binding reduction in the thalamus was associated with tremor scores [87]. However, another study found no differences between 17 PD patients and 11 controls in ^11^C-yohimbine binding across groups[88]. Further investigation of the noradrenergic system in vivo through molecular neuroimaging is needed, although currently limited by the availability of tracers, which is restricted to specialized research PET centres.

When specific noradrenergic tracers had not yet been developed, integrity of LC was investigated with ^18^F-DOPA PET. ^18^F-DOPA enters cathecolaminergic cells such as the LC noradrenergic neurons and is metabolized by AADC. Metabolic activity reductions were shown in the LC with advancing disease, while values comparable to those of controls were shown in the early stages [89, 90]. Finally, despite the uneven and sometimes conflicting results across studies, there is substantial neuropathological evidence that the other lower brainstem noradrenergic regions (A1-A5, A7) are involved in PD [18, 91, 92]. However, there is no direct MRI or PET evidence specifically showing degeneration in these regions, largely because these nuclei are extremely small and difficult to investigate with current clinical imaging techniques.

## Clinical Correlates of Noradrenergic Dysfunction in PD

In vivo investigation of the status of the central noradrenergic system has led to a greater understanding of the pathophysiological implications of noradrenergic dysfunction in PD. Disruption of noradrenergic transmission is mainly associated with cognitive impairment. However, the contribution to other manifestations has also been investigated, such as motor, sleep, neuropsychiatric and autonomic symptoms. In the following paragraphs, these clinical associations will be reviewed.

### Cognitive Impairment

Cognitive impairment has been the most investigated manifestation related to noradrenergic system degeneration in PD. Neuromelanin LC MRI-associated signal was shown to be reduced and associated with declining cognitive function in PD patients in several studies. [39, 78, 79, 93–97] However, heterogeneity exists in the level and significance of the reduction of LC neuromelanin in PD compared to controls. This is partly explained by the finding that LC involvement is likely heterogeneous in PD and associated with cognitive functioning. Indeed, reduced LC neuromelanin signal is reduced in PD with mild cognitive impairment (MCI) [98] and dementia[96], and is associated with worse cognitive performance across several domains, including attention, memory and spatial orientation [93]. Interestingly, in PD, NET availability within the LC, estimated through ^11^C-MeNER PET, is also positively correlated with z-scores of global functioning [39]. A summary of the studies that have investigated LC degeneration in PD and its effect on cognitive functions is reported in Table 1.

**Table 1:** Summary of the main in vivo studies investigating noradrenergic-related cognitive and neuropsychiatric function in PD

Author, year	Technique	Cohort	Age	Disease duration	MDS-UPDRS III	Findings
Sommerauer et al., 2018	3 T MRI (2D T1 TSE) ^11^C-MeNER PET	14 PD (RBD-) 16 PD (RBD +) 12 HC	65.4 ± 9.0 67.7 ± 9.3 67.3 ± 6.3	5.0 ± 3.6 7.6 ± 4.6 -	32.6 ± 11.9 (off) 39.3 ± 10.5 (off) -	LC MRI signal was significantly reduced in both PD groups compared to HC. ^11^C-MeNER binding was significantly reduced in PD in thalamus, hypothalamus, red nucleus, LC, raphe nuclei (with greater reductions in those RBD +). LC ^11^C-MeNER binding was associated with global cognitive z-score in PD. MRI LC signal was not associated with global cognitive z-score
Li et al., 2019	3 T MRI (2D T1 FSE)	48 PD-NC 23 PD-MCI 32 HC	60.40 ± 8.37 64.57 ± 7.37 63.44 ± 7.43	1.52 ± 1.19 1.81 ± 1.78	18.83 ± 7.55 16.96 ± 7.56 (drug-naïve)	Only PD-MCI group showed significantly reduced LC signal compared to HC. Significant association shown between reduced LC signal and worse scores in trail making test
Prasuhn et al., 2021	3 T MRI (2D dual GRE)	47 PD 53 HC	68.0 ± 8.3 70.6 ± 6.3	7.4 ± 4.8 -	27.5 ± 9.8 (on) -	LC signal significantly lower in PD compared to HC LC signal (but not substantia nigra signal) was associated with several neuropsychological test scores (attention, memory, spatial orientation); no association with motor scores
O’Callaghan et al. 2021	7 T MRI (3D MT-TFL)	19 PD 26 HC	67.11 ± 7.05 65.35 ± 5.32	4.15 ± 1.72 -	28.42 ± 11.60 (on) -	LC signal was reduced in caudal LC in PD compared to HC. Response inhibition significantly improved after atomoxetine (noradrenaline reuptake inhibitor) and the improvement is associated with reduced LC signal (greater reduction, greater improvement)
Zhou et al., 2021	3 T MRI (2D T1 TSE) rs-fMRI	94 PD 68 HC	60.64 ± 8.69 60.75 ± 7.41	3.92 ± 2.49 -	17.23 ± 10.62 (off) -	LC signal was significantly reduced in PD compared to HC and associated with MoCA scores. Reduced LC signal was associated with reduced connectivity parameters across cognitive networks
Madelung et al., 2022	7 T MRI (3D MT-GRE)	42 PD 24 HC	67.5 (38–84)* 71.5 (43–80)*	3.66 (0.25–17.3)* -	23.4 ± 9.27 (on) -	Only right LC signal was significantly reduced in PD compared to HC, more pronounced in the middle and caudal parts. An association between reduced middle-caudal LC signal and apathy scores was shown
Ye et al., 2022	7 T MRI (3D MT-TFL)	25 PD 14 PSP 24 HC	65.5 ± 5.5 69.7 ± 7.7 67.4 ± 7.4	5 ± 3.05 4.24 ± 2.68	28.36 ± 11.98 (on) 33.07 ± 6.96 -	LC signal was reduced in caudal LC in PD (and PSP) compared to HC. LC voxelwise analysis (across both groups) found significant associations between reduced LC signal clusters and lower MoCA scores
Kim et al., 2024	^18^F-FP-CIT PET	28 PD	61.9 ± 8.01	1.54 ± 0.79	25.25 ± 9.10 (off)	Subjects were scanned at baseline and after three years. FP-CIT binding was quantified in posterior putamen, dorsal raphe and LC. Lower raphe and LC binding was associated with lower MoCA scores
Wang et al., 2024	3 T (3D MT-GRE) DTI	71 PD 26 HC	61.48 ± 9.72 62.73 ± 8.56	3.00 (2.0–6.0)*	26.45 ± 10.81 (off) -	LC signal was significantly reduced in PD compared to HC, a reduction that was retained in PD with MCI and dementia, not in those with normal cognition. Diffusion along the perivascular space (ALPS) index, an indirect marker of glymphatic function, was reduced in PD and associated with LC signal and MoCA score
Wiesman et al., 2025	3 T (2D T1 FSE) EEG, MEG	58 PD 27 HC	63.02 ± 8.13 65.82 ± 9.83	6.17 ± 4.94	32.80 ± 15.20 (on)	LC signal was significantly reduced in PD compared to HC. Reduced LC signal was associated with reduced EEG alpha activity in frontal-motor regions, which is in turn associated inversely with attention scores

2D: 2 dimensional; DTI: diffusion tensor imaging; EEG: electroencephalography; FSE: fast spin echo; GRE: gradient recalled echo; HC: healthy controls; LC: locus coeruleus; MCI: mild cognitive impairment; MEG: magnetoencephalography; MT-FTL: magnetisation transfer weighted turbo flash; NC: normal cognition; PD: Parkinson’s Disease; PSP: Progressive Supranuclear Palsy; TSE: turbo spin echo;

Age, disease duration and MDS-UPDRS III values are n ± standard deviation unless otherwise indicated. *median (interquartile range)

### Neuropsychiatric Symptoms

LC activity influences not only cognitive functions but also behaviour. As an example, the noradrenergic system modulates detection of stimuli and behavioural reorienting. Response inhibition, i.e. action cancellation and attention reorientation towards salient stimuli, is critical for this task and is mediated by the noradrenergic system activity on a fronto-striatal network. Inhibition of responses towards non-salient stimuli is impaired in PD, a behavioural aspect also related to impulsivity. A series of studies explored the effect on noradrenergic system degeneration on response inhibition and found that lower LC neuromelanin signal was associated with greater impairment of response inhibition in both PD and progressive supranuclear palsy (Richardson Syndrome) [99]. Furthermore, the administration of atomoxetine, a noradrenaline reuptake inhibitor which increases extracellular noradrenaline levels in the prefrontal cortex, significantly improved response inhibition deficits, with greater improvements in patients with lower neuromelanin LC signal [94].

Reduced LC neuromelanin signal was also associated with depression (left LC reduction) and apathy (right LC reductions), in two separate studies [75, 78]. Furthermore, a PET study with [^11^C]RTI-32, a ligand for both DAT and NET, has shown specific loss of dopamine and noradrenaline innervation in the LC and in several regions of the limbic system in PD patients with depression [100]. Detailed findings of some of these studies are also shown in Table 1 [78, 94].

### Sleep Disorders

RBD is the most common sleep disturbance associated with LC degeneration in PD. Several studies reported that the presence of RBD in PD is associated with a greater degree of LC neuromelanin MRI signal loss [39, 69, 77]. Furthermore, some studies showed that the extent of LC neuromelanin loss is associated with the temporal amount of REM sleep without atonia in PD with RBD[69] and with reported RBD symptoms[98, 101]. Interestingly, these associations have also been reported in studies involving participants with isolated RBD [80, 102].

Accordingly, a PET/MRI study showed an inverse association between LC NET availability as measured by ^11^C-MeNER PET and the amount of REM sleep atonia in PD with polysomnography-proven RBD [39]. However, in the same study the association with LC MRI signal was not found.

Although the LC is involved in the complex circuitry that regulates REM sleep, the primary generator of RBD sleep atonia likely lies elsewhere, i.e. in the glutamatergic sublaterodorsal nucleus, and the degeneration of GABAergic inhibitory neurons in the ventral medulla could be the trigger for atonia loss [103].

Evidence regarding possible associations between LC degeneration may and other sleep disorders in PD is scarce, and, therefore, this could an area of further investigation in the future.

### Motor Manifestations

While motor manifestations can be largely attributed to dopamine deficiency and an abnormally functioning motor network, noradrenaline has emerged as a contributor in some motor signs of PD. The effect of the noradrenergic system on tremor is intuitive given the worsening effect of cognitive stress and the ameliorating effect of brain-penetrant non-selective beta-blockers such as propranolol [104]. Several studies suggest an effect of noradrenaline on tremor manifestation in PD. One ^11^C-MeNER PET study found preserved noradrenergic NET availability in tremulous patients in all subcortical areas investigated, except for the raphe nuclei, where it was significantly decreased. Conversely, non-tremulous PD patients showed significantly reduced NET availability in all areas compared to controls. Furthermore, rest tremor severity (in all patients) was positively associated with NET availability in median raphe, thalamus and red nucleus [86]. Interestingly, no associations with rigidity or bradykinesia scores were found. While the cause of selective reduction of NET in median raphe of tremulous patients is unclear, the authors speculate that a pathophysiological connection with serotonergic transmission may exist. Indeed, noradrenergic innervation of raphe nuclei is topographically organized and promotes serotonergic transmission. Therefore, a reduction of noradrenergic input to the raphe may also disrupt serotonergic transmission.

Notably, a reduction of serotonergic transporter binding within the raphe is associated with worse rest tremor scores [105, 106]. However, the pathophysiological link between noradrenergic and serotonergic dysfunction in PD tremor is currently speculative, as direct studies on the two systems are lacking.

Some fMRI studies investigated whether proxies of noradrenergic activation (i.e. heart rate and pupil diameter) were associated with tremor manifestation and tremulous activity within the cerebello-thalamo-cortical network, which is currently known as the primary source of tremor oscillations in the brain. One study identified two main mechanisms by which tremor is increased by cognitive stress: a bottom-up mechanism driven by the ascending arousal system (which includes noradrenergic transmission) and a top-down “stress response” cortical network; both mechanisms converge onto the cerebello-thalamo-cortical network to enhance its tremor-related activity [107]. Another recent study from the same group also highlighted how propranolol reduced tremor amplitude by reducing tremor-related activity specifically in the motor cortex (but not in the other nodes of the cerebello-thalamo-cortical network) [108]. In Table 2 a summary of studies investigating the effect of the noradrenergic system on PD tremor is provided [83, 86, 107–110].

**Table 2:** Summary of the main in vivo studies investigating noradrenergic dysfunction and tremor in PD

Author, year	Technique	Cohort	Age	Disease duration	MDS-UPDRS III	Findings
Nahimi et al., 2018	^11^C-MeNER PET	15 PD 10 HC	64.8 ± 8.8 65.7 ± 8.2	9.9 ± 2.8 -	42.3 ± 7.5 (off) -	Tracer uptake is significantly reduced in red nucleus and thalamus of PD patients. Tremor occurrence was associated with higher thalamic tracer binding in PD
Dirkx et al., 2020	3 T MRI (fMRI)	33 PD T +	66 ± 9.7	3.1 ± 2.4	42.0 ± 14.4 (off) (MDS-UPDRS total)	Pupil diameter and heart rate were recorded together with EMG tremor activity during rest and cognitive load (mental arithmetic task), in a fMRI scan. Cognitive load was associated with increase in tremor, heart rate and pupil diameter. Dynamic causal modelling analysis of fMRI data suggested that cognitive load increases tremor in two ways: by influencing tremor-related activity in the thalamic VLpv likely through the ascending arousal system (including noradrenaline), and by strengthening the cerebello-thalamo-cortical network connectivity through cortical activity
Kinnerup et al., 2021	^11^C-MeNER PET	37 PD T- 28 PD T + 28 HC	68 ± 8 65 ± 9 66 ± 7	6.2 ± 3.7 5.9 ± 4.1	NA NA	Tracer uptake was significantly higher in LC and thalamus of tremulous patients. In tremulous patients LC tracer uptake was similar to controls, while in the raphe was significantly reduced. In non-tremulous patients, tracer uptake was significantly reduced in all regions compared to controls. Rest tremor severity (in all patients) was associated with higher NET availability in median raphe, thalamus and red nucleus
Dirkx et al., 2023	3 T MRI (fMRI)	40 PD T +	63.3 ± 3.2	2.9 ± 2.19	44.7 ± 17.4 (off) (MDS-UPDRS total)	A “brain connectivity integration” analysis revealed that cognitive load increases cerebral integration, also in the cerebello-thalamo-cortical network. Furthermore, cerebral integration increases significantly about 13 s *before* the tremor episodes (and covaries with subsequent tremor amplitude), but was not increased *during* the tremor episode. Meanwhile, pupil diameter and heart rate covaried with tremor amplitude. These findings suggest that within- and between-networks brain connectivity increases before tremor, and the arousal system amplifies this mechanism
van der Heide et al., 2025	3 T MRI (fMRI)	27 PD T +	61.4 ± 8.2	4.2 ± 2.9	28.8 ± 10.8 (off)	This was a cross-over double-blind study investigating the effect of propranolol 40 mg single dose vs placebo on tremor and cerebral tremor-related activity. Propranolol reduced rest and postural tremor during both rest and cognitive task; furthermore, propranolol reduced tremor-related activity in the contralateral motor cortex, but not in the remaining nodes of the cerebello-thalamo-cortical network
Sun et al. 2025	3 T MRI (2D MT-GRE; fMRI)	48 PD-TD 72 PD-PIGD 83 HC	60.1 ± 8.6 60.1 ± 9.5 59.3 ± 8.3	53.8 ± 47.9* 62.3 ± 57.7	22.98 ± 10.19 (off) 27.76 ± 13.35 (off) -	LC neuromelanin signal significantly reduced on more affected side of both TD and PIGD compared to controls, and in PIGD compared to TD. In PD-TD rest tremor scores were associated with preserved LC neuromelanin signal. Effective connectivity between LC and nodes of the cerebello-thalamo-cortical network was increased in TD compared to PIGD but this measure was not correlated to tremor. Effective connectivity between LC and motor cortex was associated with rest tremor scores in TD patients

GRE: gradient recalled echo; MT: magnetisation transfer PD: Parkinson’s Disease; NA: not available; PET: Positron Emission Tomography; PIGD: Postural Instability Gait Difficulty; T + : Tremulous PD; T-: non-tremulous PD; TD: tremor dominant. *disease duration expressed in months

The effect of noradrenergic system degeneration on the other cardinal signs of PD has been hypothesized but evidence is scarcer [87]. One study found reduced NET availability in the motor cortex of PD patients compared to controls, a reduction that was more pronounced in patients with Hohen&Yahr stage > 2. However, this could also represent an epiphenomenon of a more advanced disease [85].

A few recent studies investigated the role of noradrenergic dysfunction in freezing of gait (FOG). In one study, PD with FOG showed significantly reduced LC neuromelanin signal, with similar SN neuromelanin, compared to PD without FOG and controls. Furthermore, in PD with FOG, greater severity of some LC neuromelanin parameters was associated with worse reported FOG symptoms [111]. Another study with ^11^C-MeNER PET showed a NET reduction in the right thalamus of PD patients experiencing FOG in the OFF state only, and this reduction was in turn associated with more severe reported FOG symptoms. Interestingly, patients with FOG in both OFF and ON states, did not show NET reductions, similarly to patients without FOG [112]. When interpreting these findings, it should be acknowledged that FOG likely has a complex pathophysiology involving transient network disruption in multiple networks and in multiple neurotransmitter systems such as dopamine and acetylcholine [113]. The complexity of the FOG phenomenon is highlighted by another study in which the authors investigated the effect of an anxiety-induced situation on FOG [114]. Through fMRI coupled with pupil diameter recordings, patients navigated a threatening and a non-threating virtual reality situation. The threatening situation provoked more freezing and was associated with increased connectivity between the motor and cognitive networks and reduced connectivity within the limbic network; this was in turn associated with increased pupil dilation. Interestingly, fMRI showed an overall increase in brain networks integration during the threatening condition, a phenomenon that resembles Dirkx et al.’s findings regarding the increase in brain networks integration preceding tremor episodes (and pupil diameter increase) [109]. Noradrenergic-driven brain networks integration has also been shown previously in healthy controls[115, 116] and, therefore, it appears to be a physiologic mechanism that could be disrupted by PD pathology.

### Autonomic Manifestation and Peripheral Noradrenergic Dysfunction

The LC is part of the central network controlling autonomic function. Its noradrenergic transmission increases peripheral sympathetic activity and decreases parasympathetic activity. Physiologically, peripheral sympathetic activity is critical for cardiovascular regulation, thermoregulatory responses, bronchial dilation, pupil dilation, contraction of the internal urethral sphincter and relaxation of bladder detrusor muscle, among others.

In healthy subjects, higher LC neuromelanin MRI signal is associated with heart rate-derived parameters of sympathetic activation and parasympathetic inhibition, the latter influence exerted directly on vagal nuclei [117, 118]. It could be then hypothesized that LC degeneration may also impair cardiac and baroreflex responses in PD. One study found an association between systolic blood pressure drop and left LC neuromelanin loss [78]. However, two studies did not find similar associations [39, 101]. Nonetheless, a study with ^11^C-MeNER PET found an association between NET availability in the LC and hypothalamus and systolic and diastolic blood pressure drop. However, this finding was significant only in a pooled analysis that included PD (with and without RBD) and controls [39].

Pathological involvement of the peripheral sympathetic system likely plays an important role in autonomic dysfunction. Alpha synuclein pathology is indeed found in the heart sympathetic terminals and in the stellate ganglion[119, 120], and recently a sympathetic route of spreading PD pathology has been hypothesized [121]. Furthermore, in vivo cardiac denervation is a usual finding in PD, dementia with Lewy bodies and their prodromal manifestations [122–125].

Finally, it is intuitive that autonomic dysfunction in PD may arise both from the central autonomic network dysfunction, which involves the LC, and the peripheral dysfunction of sympathetic and parasympathetic terminals. A more detailed description of autonomic dysfunction pathophysiology in PD is beyond the scope of this article and can be found in *Chen *et al.[126]

## Treatment of Noradrenergic Dysfunction

Despite the development of specific in vivo neuroimaging techniques that significantly increased the knowledge about central noradrenergic dysfunction, targeted treatments are still limited. Droxidopa is the only United States (US) Food and Drug Administration (FDA) approved drug targeting the noradrenergic system with a specific indication for PD, i.e. for the treatment of neurogenic orthostatic hypotension. In a randomized controlled trial, Droxidopa, a modified aminoacidic precursor of noradrenaline, improved symptomatic orthostatic hypotension, with an associated increase in standing systolic blood pressure [127]. Droxidopa was also approved in Japan but is not available in Europe.

Venlafaxine, a reuptake inhibitor of serotonin and noradrenaline (SNRI), was efficacious for PD depression when compared to placebo and paroxetine in a randomized controlled-trial [128]. Duloxetine showed a similar favourable profile in PD depression; however, evidence only comes from an open label study [129].

Atomoxetine and methylphenidate are respectively selective norepinephrine and norepinephrine-dopamine reuptake inhibitors indicated for the treatment of Attention-Deficit/Hyperactivity Disorder (ADHD). As discussed in the “Neuropsychiatric symptoms” paragraph, atomoxetine provided improvements in response inhibition, an aspect that is linked to impulsivity in PD, and these improvements were greater in subjects with more severe LC neuromelanin signal reductions [94, 99]. A pilot open label trial suggested benefit in executive dysfunction from atomoxetine (100 mg/daily) in 12 PD subjects [130]. Another randomized clinical trial in 55 PD patients showed a mean 1.3 points improvement in Mini Mental State Examination (MMSE) (but did not meet its primary endpoint, i.e. improvement in depression) [131]. Another clinical trial involving 30 PD participants with MCI receiving either atomoxetine (up to 80 mg/daily) or placebo showed no benefit in a battery of executive function tests, but showed improvement trends in subjective attention and cognition scores.

Methylphenidate was trialled for PD-related gait impairment with no benefit and actually led to a worsening of motor scores in treated patients [132]. Conversely, in another study in PD patients with subthalamic deep brain stimulation, methylphenidate improved gait hypokinesia and freezing [133].

Clonidine is an α_2_-adrenergic receptor agonist with current indications for hypertension, ADHD and tics in Tourette syndrome. While the effect of clonidine is usually attributed to noradrenergic transmission inhibition by activation of α_2_-adrenergic autoreceptors on noradrenergic terminals, higher doses may engage post-synaptic receptors with enhancing effects [134, 135]. In ADHD and Tourette syndrome, clonidine provides benefits on hyperactivity and impulsivity likely by impacting the top-down control role of the prefrontal cortex [136, 137]. A recent phase 2b clinical trial with clonidine 75 μg twice daily was conducted to evaluate its effect on impulse-control disorder (ICD) in PD. Clonidine was well tolerated and led to a non-significant reduction in ICD scales scores than placebo. The study, however, was not conclusive to evaluate efficacy, and a phase 3 trial would be needed for that objective [138].

Overall, evidence for noradrenergic modulation in PD is growing and showing potential in several PD manifestations, especially non-motor symptoms. Larger studies with stratified PD populations based on symptom manifestation, and possibly a neuroimaging surrogate of noradrenergic function, are needed. Furthermore, the effect of propranolol on PD tremor should not be disregarded and could be further investigated.

## Conclusions

Degeneration and dysfunction of the noradrenergic system in PD can now be investigated in vivo through neuromelanin MRI and PET with noradrenergic ligands. LC Neuromelanin signal loss and axonal terminal loss are found in PD, with more severe involvement in non-tremulous PD and in more advanced motor stages. Conversely, tremulous PD shows more preserved noradrenergic system neuroimaging markers.

Physiological functions of the noradrenergic system are pervasive on behaviour, cognition, movement, arousal and sleep. Hence, its dysfunction in PD contributes to several manifestations such as cognitive decline, apathy, impulsivity, RBD, tremor, freezing of gait and autonomic dysfunction. All these manifestations are indeed less responsive to dopaminergic medications and some have limited pharmacological interventions available.

Pharmacological targeting of the noradrenergic system is challenging, owing to the very wide distribution and functions of the peripheral sympathetic and central noradrenergic system receptors. Nonetheless, development or repurposing of drugs modulating the noradrenergic system appears critical for treating noradrenergic-related manifestations in PD.

Overall, future studies on pathophysiological aspects of noradrenergic dysfunction and clinical trials targeted at noradrenergic modulation have the potential to improve several PD manifestations with limited responsiveness to dopaminergic treatment, such as tremor, freezing of gait, cognitive, behavioural and autonomic symptoms.

## Key References

- Doppler CEJ, Kinnerup MB, Brune C, Farrher E, Betts M, Fedorova TD, et al. Regional locus coeruleus degeneration is uncoupled from noradrenergic terminal loss in Parkinson’s disease. Brain; 2021;144:2732–44. 10.1093/brain/awab236
  - This multimodal neuromelanin and 11C-MeNER PET study shows decoupling of LC-neuromelanin signal loss and subcortical nuclei axon terminal loss, with the latter exceeding by 10-20% the former in PD compared to controls. This suggests a dying-back mechanism in vivo in PD in the noradrenergic system.
- Dahl MJ, Mather M, Düzel S, Bodammer NC, Lindenberger U, Kühn S, et al. Rostral locus coeruleus integrity is associated with better memory performance in older adults. Nat Hum Behav. Springer US; 2019;3:1203–14. 10.1038/s41562-019-0715-2.
  - This MRI study shows that in healthy older adults while LC integrity is related to episodic memory performance, SN-VTA integrity is linked to working memory performance.
- Laurencin C, Lancelot S, Brosse S, Mérida I, Redouté J, Greusard E, et al. Noradrenergic alterations in Parkinson’s disease: a combined 11C-yohimbine PET/neuromelanin MRI study. Brain; 2024 ;147:1377–88. 10.1093/BRAIN/AWAD338
  - This study used ^11^C-yohimbine PET to investigate noradrenergic terminals and LC neuromelanin MRI. Reduction of α2 receptor availability in the thalamus was associated with tremor, while a reduction in the putamen, the insula and superior temporal gyrus was associated with anxiety.
- Wiesman AI, Madge V, Fon EA, Dagher A, Collins DL, Baillet S. Associations between neuromelanin depletion and cortical rhythmic activity in Parkinson’s disease. Brain; 2025;148:875–85. 10.1093/BRAIN/AWAE295
  - This multimodal neuromelanin and high-density EEG study showed that LC neuromelanin MRI signal loss was associated with reduced EEG alpha activity in frontal-motor regions and this was in turn associated with attention scores.
- van der Heide A, Wessel M, Papadopetraki D, Geurts DEM, van Prooije TH, Gommans F, et al. Propranolol Reduces Parkinson’s Tremor and Inhibits Tremor-Related Activity in the Motor Cortex: A Placebo-Controlled Crossover Trial. Ann Neurol; 2025;97:741–52. 10.1002/ANA.27159
  - This cross-over randomized clinical trial showed ameliorating effect of single-dose propranolol 40 mg on rest and postural tremor and reduction of fMRI tremor-related activity in the contralateral motor cortex.

## Data Availability

No datasets were generated or analysed during the current study.
